# A study protocol for risk stratification in children with concussion (RSiCC): Theoretical framework, design, and methods

**DOI:** 10.1371/journal.pone.0306399

**Published:** 2024-07-18

**Authors:** Karin Reuter-Rice, Amanda N. Fitterer, Peter Duquette, Qing Yang, Anushka K. Palipana, Daniel Laskowitz, Melanie E. Garrett, Margaret Fletcher, Julia Smith, Lynn Makor, Gerald Grant, Kristen Ramsey, O. Josh Bloom, Allison E. Ashley-Koch

**Affiliations:** 1 Duke University School of Nursing, Durham, North Carolina, United States of America; 2 Department of Pediatrics, Duke University School of Medicine, Durham, North Carolina, United States of America; 3 Department of Neurosurgery, Duke University School of Medicine, Durham, North Carolina, United States of America; 4 Department of Physical Medicine & Rehabilitation, Univeristy of North Carolina at Chapel Hill, School of Medicine, Chapel Hill, North Carolina, United States of America; 5 Department of Neurology, Duke University School of Medicine, Durham, North Carolina, United States of America; 6 Duke University School of Medicine, Duke Molecular Physiology Institute, Durham, North Carolina, United States of America; 7 Department of Public Instruction, State of North Carolina, Office of Exceptional Children, Raleigh, North Carolina, United States of America; 8 Duke University Health System, Carolina Family Practice and Sports Medicine, Carolina Sports Concussion Clinic, Cary, North Carolina, United States of America; PLOS: Public Library of Science, UNITED KINGDOM OF GREAT BRITAIN AND NORTHERN IRELAND

## Abstract

Research shows that one in five children will experience a concussion by age 16. Compared to adults, children experience longer and more severe postconcussive symptoms (PCS), with severity and duration varying considerably among children and complicating management of these patients. Persistent PCS can result in increased school absenteeism, social isolation, and psychological distress. Although early PCS diagnosis and access to evidence-based interventions are strongly linked to positive health and academic outcomes, symptom severity and duration are not fully explained by acute post-injury symptoms. Prior research has focused on the role of neuroinflammation in mediating PCS and associated fatigue; however relationship between inflammatory biomarkers and PCS severity, has not examined longitudinally. To identify which children are at high risk for persistent PCS and poor health, academic, and social outcomes, research tracking PCS trajectories and describing school-based impacts across the entire first year postinjury is critically needed. This study will 1) define novel PCS trajectory typologies in a racially/ethnically diverse population of 500 children with concussion (11–17 years, near equal distribution by sex), 2) identify associations between these typologies and patterns of inflammatory biomarkers and genetic variants, 3) develop a risk stratification model to identify children at risk for persistent PCS; and 4) gain unique insights and describe PCS impact, including fatigue, on longer-term academic and social outcomes. We will be the first to use NIH’s symptom science model and patient-reported outcomes to explore the patterns of fatigue and other physical, cognitive, psychological, emotional and academic responses to concussion in children over a full year. Our model will enable clinicians and educators to identify children most at risk for poor long-term health, social, and academic outcomes after concussion. This work is critical to meeting our long-term goal of developing personalized concussion symptom-management strategies to improve outcomes and reduce disparities in the health and quality of life of children.

## Introduction

Concussions occur at an alarming rate among U.S. schoolchildren, with approximately one in five experiencing a concussion by age 16 [1]. Compared to adults, children experience longer and more severe postconcussive symptoms (PCS) [2–4]. Severity and duration of PCS, however, vary considerably among children, complicating clinical care and return to learn and play decisions [5, 6]. Although PCS diagnosis and access to evidence-based return-to-health and -school interventions are strongly linked to positive health and academic outcomes [7], clinical models to identify children who are at high risk for persistent PCS are lacking.

Concussions are on the rise due in part to better awareness for this injury. Reports indicate that over the past decade, annual emergency department visits by children for concussion have increased by 50% [8, 9], representing a major public health burden. Societal costs have been estimated to be as high as $1 billion annually [8, 9] in the U.S. Persistent physical, emotional, and cognitive symptoms result in increased school absenteeism, social isolation, and psychological distress [10, 11]. Acute symptom severity alone, however, is a poor prognostic indicator of clinical outcomes. Symptom severity immediately postconcussion does not explain why at least 25% of children still experience PCS after one year [2–4], or why children who may appear asymptomatic still report academic and social challenges months after concussion [12]. To identify which children are at high risk for persistent PCS and poor health, academic, and social outcomes, research tracking PCS trajectories and describing school-based impacts across the entire first year postconcussion is critically needed.

Pediatric concussion research has focused primarily on the PCS of headache, dizziness, and cognitive difficulties [13]. Yet, 73% of children with PCS also report experiencing persistent fatigue [14, 15]. Fatigue diminishes physical or mental capacity and might, in the absence of more overt concussion symptoms, account for negative social and academic outcomes [16, 17]. Studies have found that one third of patients with concussion complain of severe fatigue at six months, while 40% complain of chronic fatigue one year postconcussion [18, 19]. Importantly, severity of fatigue early postconcussion has been linked to long-term PCS [4, 20]. Although possible causes of fatigue in children with concussion have been suggested—including postconcussion biologic changes, psychological problems, sleep disturbances, and pubertal changes—conclusive findings are lacking [13]. Because concussion-related fatigue is difficult to quantify, it often goes unrecognized and untreated thereby impacting quality of life.

Multiple factors interact to influence concussion recovery in children, including premorbid characteristics, mechanism of injury, age, sex, and factors like race, ethnicity, and socioeconomic status (SES) that contribute to health disparities more broadly [3, 4, 21]. However, there are significant knowledge gaps regarding recovery from pediatric concussion [3]. Recovery is variable, and no single factor or assessment tool has been identified that predicts symptom resolution or outcome [22–24]. Guidance is lacking regarding timing of interventions or progression of return to academic activities. Further, evidence is insufficient to identify a particular biomarker for the prognosis of concussion in children. Most concussion therapy recommendations do not consider factors such as race/ethnicity or SES and are primarily based on sports-related injuries, neglecting other causes of concussion [3].

This project will define novel PCS trajectory typologies in a racially/ethnically diverse population of 500 children with concussion (11–17 years, near equal distribution by sex), identify associations between these typologies and patterns of inflammatory biomarkers, and develop a PCS risk stratification model. The NIH-SSM complex symptom collection will include postconcussion symptom reporting along with patient-reported outcomes (PROs) to explore the patterns of fatigue and other physical, cognitive, and emotional responses to concussion in children over a full year. Additionally, we will collect biologic samples over six time points throughout the year. We will use a novel statistical approach—multivariate growth mixture modelling (GMM)—to identify distinct PCS trajectory typologies. Combining these trajectory typologies with biomarker patterns and demographic and injury characteristics, we will develop a model that enables clinicians and educators to identify children most at risk for poor long-term health, social, and academic outcomes after concussion. To gain unique insight into the effects of PCS, including fatigue, on longer-term academic and social outcomes, we will also collect school-based data over the entire year. This work is essential to meeting our long-term goal of developing personalized symptom-management strategies to improve outcomes and reduce disparities in the health and quality of life of children with concussions.

### Specific purposes

**Aim 1.** Identify specific trajectories of fatigue and other PCS in children.
**Aim 2.** Identify potential inflammatory cytokines and genetic variants associated with PCS.
**Aim 3.** Develop a risk stratification model using demographics, concussion characteristics, inflammatory biomarker patterns, and PCS trajectory typologies to identify children at high risk for persistent PCS.
**Aim 4.** Describe the impact of concussion on self and schooling over the first year following injury.

### Conceptual model

**Fig 1:**
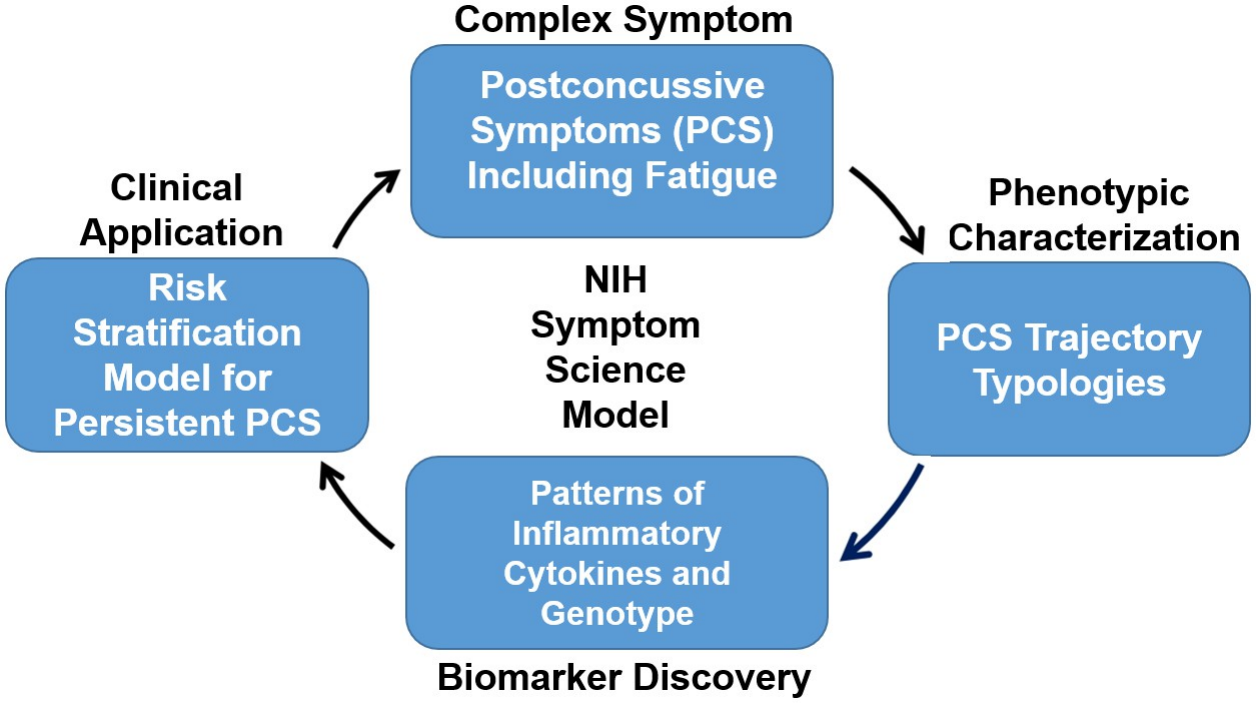
RSiCC Study Framework adapted from NIH-SSM. The study framework combines injury characteristics, medical and psychosocial histories, patient reported outcome surveys, and biologic signatures to develop a model to stratify risk for prolonged PCS in pediatric concussion.

This project’s framework (Fig 1) is adapted from the NIH’s symptom science model (NIH-SSM) [25], which incorporates complex symptoms, phenotypic characteristics, biomarker discovery and clinical translation. NIH-SSM describes the sequence employed to investigative symptom science research. It begins with the identification of a symptom, or cluster of symptoms, followed by characterization of phenotypes. Importantly, the model provides an opportunity to include biomarker discovery, which allows for more clinically robust symptom translation. To date, use of inflammatory biomarkers (cytokines) in concussion research has focused primarily on evaluation of acute symptom severity [26–28], while the relationship between inflammatory biomarkers and longitudinal PCS severity remains poorly defined. Expanding this focus to examine the longitudinal patterns of inflammatory responses in children with concussions could provide the data needed to develop and test anti-inflammatory treatment protocols to improve physical and mental health outcomes. Moreover, genetic variants (ie. single nucleotide polymorphisms [SNPs]) involved in mediating inflammation may also influence inflammatory processes in brain injury and PCS [29–34]. A growing body of evidence suggests that such variants confer neuroprotection or vulnerability after brain injury [12, 35–38]. Yet studies examining biomarkers and genetic variant profiles in children with concussion are lacking [29]. Measuring inflammatory biologic responses for one year postconcussion will provide novel opportunities to develop targeted therapies for those symptomatic children.

## Methods

### Design and sample

This prospective, longitudinal cohort study will follow participants over a one-year period. Participants will be recruited from four concussion clinics across two health systems in the Triangle region of North Carolina. Target accrual for this study is 500 subjects. Based on historical patient demographic data from the study sites, it is anticipated that the sample will comprise 55% male and 45% female, 80% Non-Hispanic and 20% Hispanic, 50% Caucasian/White, 40% African American/Black, and 10% of all other races, and 40% low socio-economic status.

The study was approved by the Duke University Institutional Review Board with a single IRB to include all study sites. Recruitment began in May 2023 and is anticipated to continue through February of 2028.

### Eligibility criteria

Subjects will meet the inclusion criteria: aged 11 to 17 years at the time of enrollment, diagnosed with mTBI/concussion that occurred within the prior seven days, Glasgow Coma Scale (GCS) Score between 13–15, and English speaking. Exclusion criteria include: diagnosed with current moderate or severe TBI, polytrauma, nontraumatic brain injury, non-English speaking, and pregnancy.

### Promotion of project participation and retention

To minimize barriers that could limit participation of both the participant and their family (e.g. missed school or work, transportation costs, child- or elder-care of family members, or conflicting healthcare appointments) data collections will occur either in-person in the clinic or participant’s domicile with their family present or remotely via phone or videoconferencing. These trialed methods have resulted in successful data collection and helped reduce study burden. During our pilot study, videoconferencing was as successful as in-person because we could see and interact over the conferencing platform in real time.

### Data collection procedures

Data collection begins after parent consent and child assent has been obtained. Data will be collected during six study visits over the course of one year for each participant. The initial study visits may occur in person at the concussion clinic where they are receiving care for their concussion or at home after consent/assent has been obtained. Follow up visits may occur in person or via videoconferencing.

Demographics, medical history and psychosocial history will be obtained during the initial study visit through a combination of medical records review and participant interview. Demographic, medical history, and psychosocial history questionnaires are available in S1 Appendix. Each child’s concussion will be deeply characterized using their comprehensive health history to identify PCS risk factors. See S2 Appendix for the complete Concussion Characteristics data collection instrument.

#### Questionnaires

*Post Concussion Symptom Scale (PCSS)*: This 22-item scale measures severity of concussion symptoms using a 7-point Likert scale ranging from 0 (no difficulty) to 6 (severe difficulty) [39]. Pre-injury PCSS score will also be collected at the initial visit.

*PROMIS Pediatric Item Bank v2*.*0 –Fatigue*: This pediatric self-report measures fatigue symptoms in children aged 8–17 years. Its 25 calibrated items range from mild subjective feelings of tiredness to an overwhelming sense of exhaustion using a 5-point Likert scale ranging from never tired (1) to almost always tired (5) [40, 41]. Pre-injury fatigue level will also be collected at the initial visit.

*NeuroQoL Pediatric Short Forms*: Neuro-QoL^™^ measures are used in a variety of pediatric neurological conditions, including TBI [42]. Neuro-QoLs are standardized health related quality of life assessments [42]. The Pediatric Neuro-QoL is comprised of eight generic or targeted banks (anxiety, depression, anger, interaction with peers, fatigue, pain, applied cognition, and stigma) and two generic scales (upper and lower extremity function) [42]. For item banks that consist of more than ten questions, a brief version, or short-form, has been developed. Short forms with eight items each will be used in this study to minimize respondent fatigue.

- *Neuro-QoL Item Bank v1*.*0 –Pediatric Social Relations–Interaction with Peers–Short Form*: Items focus on patient-reported involvement with peers in usual social roles, activities, and responsibilities.
- *Neuro-QoL Item Bank v2*.*0 –Pediatric Cognitive Function–Short Form*: Items focus on patient-reported difficulties with basic cognitive abilities such as memory, attention, concentration, processing speed, and organization.

*Academic Performance and Self-Assessment*: The Concussion Learning Assessment & School Survey, 3^rd^ Edition (CLASS-3) measure consists of four scales (General Academic Concern, Academic Problems, School Stresses, and Academic Subjects). A cumulative score is generated for each of the four scales, which in sum can be considered as a clinical measure to assess and monitor the academic needs of a student following concussion [43].

Additional academic performance items will be added to the CLASS-3 for this study to obtain additional academic performance data. At T1–T6, we will collect self-reported loss of time in school (missed number of days), report card grades, difficulty with academic subjects (math, English, science, etc.), and time to return to learn and physical activities [15]. See S3 Appendix for the complete CLASS-3 Adapted instrument with additional items for this study.

*Biologic Measures*: Salivary samples will be used for genetic analysis at study enrollment and biomarker analyses at T1–T6. Salivary specimens are a convenient, cost-effective, and less stressful alternative to blood specimens. The literature confirms that saliva is as reliable a biospecimen as blood for the study measures [44–49]. The focus will be on inflammatory cytokines and candidate genes with a documented association with concussion, mTBI, or fatigue [50, 51]. To determine if fatigue is influenced by pubertal change, a salivary puberty marker (dehydroepiandrosterone [DHEA]) will be collected at T1–T6.

*Genotyping*: Recent evidence suggests that specific SNPs may modify outcomes in adults with mTBI [38, 52, 53]. Such associations, however, have not been investigated in children [29, 38]. Genetic variants in the 11 candidate genes listed in Table 1 will be examined for associations with PCS trajectory typologies. Included are genes that code for the inflammatory cytokines of interest (Table 2) as well as genes previously implicated in adult TBI genetic studies.

**Table 1:** Inflammatory genetic variants involved in brain injury and in fatigue.

Gene (*Gene Symbol*)
Apolipoprotein E *(APOE)*
Immunoglobulin superfamily, member-3 *(IGSF3)*
Interferon gamma *(IFN-γ)*
Interleukin-1beta *(IL-1β)*
Interleukin-6 *(IL-6)*
Interleukin-8 *(IL-8)*
Interleukin-10 *(IL-10)*
Microtubule-associated binding protein tau *(MAPT)*
Tumor necrosis factor-alpha *(TNFα)*
TNFα-induced proteins 1 *(TNFAIP1)*
TNFα-induced proteins 8 (*TNFAIP8*)

**Table 2:** Inflammatory cytokines, actions specific to the brain, and associated PCS.

Cytokine	Action	PCS
IFN-γ	Induces IL-1, TNFα secretion, and depletion of serotonin. Associated with cognitive and psychomotor dysfunction, anxiety/depression, and fatigue.	Fatigue
IL-1β	Proinflammatory cytokine associated with persistent low-grade inflammation. Affects hippocampal neurons and synaptic plasticity, inhibiting synaptic strengthening. Associated with cognitive dysfunction	Headache, fatigue, cognitive difficulties, sleep disturbance
IL-6	Proinflammatory cytokine associated with synaptic plasticity and strengthening. Affects learning/memory.	Fatigue, cognitive difficulties, sleep disturbance, depression/anxiety
IL-8	Mediator of acute inflammation that primarily targets recruitment-activation of neutrophil granulocytes and other intra/extracellular changes in response to inflammation and trauma. Affects cognition.	Cognitive difficulties
IL-10	Anti-inflammatory cytokine that inhibits synthesis of cytokines (e.g., IFN-γ, TNFα) and suppresses proinflammatory responses that promote phagocytosis. May enhance cognition.	Cognitive difficulties
TNFα	Alters synaptic strengthening, plasticity, and efficacy and decreases synaptic inhibition. Activates caspases and promotes death-signaling pathway. Affects cognitive function.	Headache, fatigue, cognitive difficulties, sleep disturbance, depression/anxiety

*Note*. IFN- γ = interferon gamma; IL = interleukin; TNFα = tumor necrosis factor alpha

### Expected statistical procedures

Descriptive statistics will be calculated for baseline demographic and concussion characteristics. All data management and descriptive analyses will be conducted in SAS 9.4. MPLUS 8.4 will be used for constructing GMMs, pattern mixture models, or other latent variable models. All p-values will be two sided.

**Aim 1.** *Identify specific trajectories of fatigue and other PCS in children over the first year postinjury to define distinct PCS trajectory typologies*. Multivariate GMM will be used to cluster 500 children with concussion into distinct typologies, based on trajectory patterns of their PCSS total score and PROMIS Fatigue symptom score over six time points (T1–T6) simultaneously [54–56]. To account for the possibility that puberty may influence the underlying trajectories, time-varying measures of puberty (DHEA) will be directly incorporated as key model parameters when defining/extracting the trajectories and identifying group membership.

We will construct models ranging from one to six classes. The selection of the optimal model and risk profiles (classes) will be determined bybalancing considerations of continuity, BIC statistics, and overall clinical utility.

**Fig 2:**
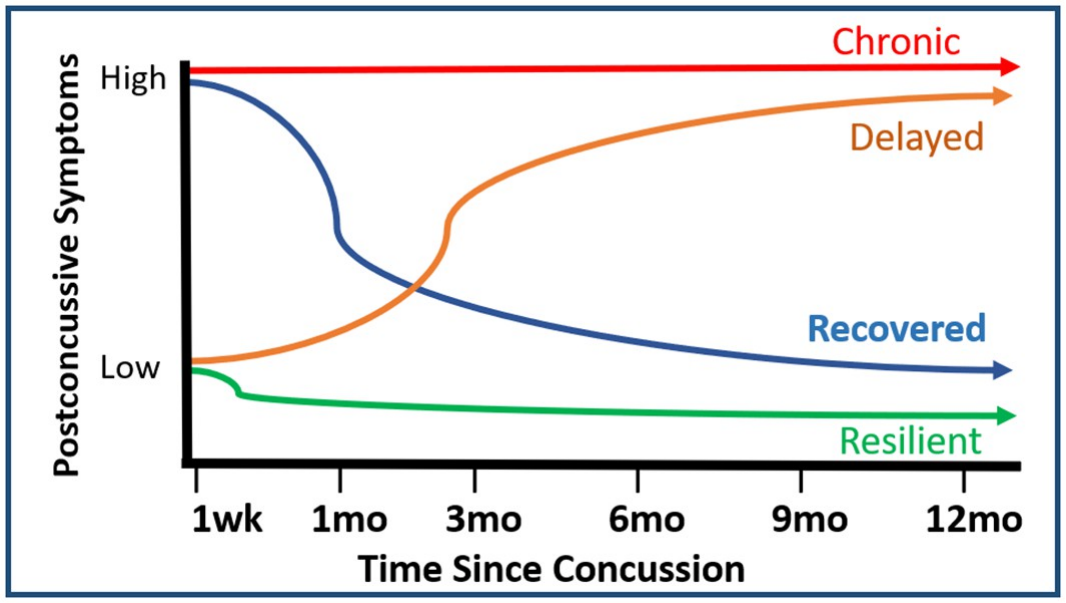
Potential PCS trajectory typologies. Children might have a high number of PCS early on that either resolve (*recovered)* or persist (*chronic)*. Or they might have a low number of PCS early on and either remain relatively asymptomatic (*resilient)* or develop additional PCS over time *(delayed)*. Adapted from Galatzer-Levy et al., 2018.

Four PCS prototypic trajectory typologies are expected: resilient, recovered, delayed, and chronic (Fig 2) [57]. To accommodate potential patient variability in the profiles of multiple symptom variables, up to six multi-trajectory groups will be examined during model development.

**Aim 2.** *Identify potential inflammatory biomarkers for PCS trajectory typologies*.

- 2a. *Determine associations between PCS trajectory typologies and inflammatory cytokines over six time points*. The levels of six inflammatory cytokines (Table 2) will be assessed over T1–T6. To assess their associations with PCS trajectory typologies, cytokine trajectories will be characterized using GMM. The modeling steps will be similar to those for Aim 1 but with cytokines. It is expected that there will be four distinct trajectory patterns for each cytokine (i.e., consistent high, consistent low, increase, and decrease). Chi-square tests will then be used to assess bivariate associations between PCS trajectory typologies and cytokine trajectory patterns. It is expected that some cytokine trajectory patterns will correlate with symptom trajectory typologies. Multiple comparisons for six inflammatory cytokines will be adjusted using Bonferroni adjustment.
- 2b. *Determine associations between variants in inflammatory genes and PCS trajectory typologies*. Single-variant analyses will be performed to evaluate approximately 10 SNPs within each of the 11 candidate genes listed in Table 1. Comparisons for all four PCS trajectory typologies will be made. However, this analysis has been powered to primarily detect differences between the resilient typology and each of the other three (chronic, delayed, and recovered) as these will be the most powerful comparisons due to expected sample sizes. Identification of genetic variants with a minor allele frequency of 25%–40% that confer a moderate risk (OR > 2) for differences in PCS trajectory typology is expected. This estimate assumes an additive genetic model and α = 4.5 x 10^−4^, which is a Bonferroni correction for a nominal p-value of 0.05 divided by 110 single-variant tests (10*11). If there are more or less than ten SNPs per candidate gene examined, the appropriate multiple testing correction will be made. Variables significantly associated with trajectory typologies will be included in the models to control for confounding. Logistic regression will be used to test for associations between SNPs and PCS trajectory typologies [58]. Covariates will include, at minimum, age, biological sex, genetic ancestry PCs, and puberty status. To correct for multiple testing of single-variant analyses and a limited number of examined SNPs (n = 110), a Bonferroni approach will be used. Depending on the distribution of sex in the PCS prospective data set, a determination will be made as to whether it is reasonable to perform exploratory sex-stratified analyses. Gene-burden tests may also be performed for each candidate gene whereby all the variants per candidate gene would be grouped rather than examining each SNP separately.

**Aim 3.** *Develop a risk stratification model using demographics*, *concussion characteristics*, *inflammatory biomarker patterns*, *and PCS trajectory typologies to identify children at high risk for persistent PCS*. Descriptive statistics will be calculated for all available variables, including demographics, concussion characteristics, cytokine patterns, genetic variants at baseline, and PCS trajectory typologies. The stratification model will be developed in a three-step process.

- Step 1: The associations between each participant’s demographic and concussion characteristics with their PCS trajectory typology will be assessed using appropriate statistical tests.statistical. Cytokine and genetic biomarkers will be chosen based on test results from Aim 2. Any biomarkers that differ between typologies with a priori p < 0.20 will be considered for the stratification model. IdentifyIdentify highly correlated predictors (r > 0.8), selecting one based on clinical relevance and interpretability.
- Step 2: Two logistic models will be built to predict likelihood of being in a higher-risk group—one model for resilient versus delayed, the other model for recovered versus chronic. All prescreened biomarkers from Step 1 will enter the variable-selection process.
- Step 3: To check the validity of the model, the bootstrap method will be used, asas it can capture all sources of model uncertainty. The overall discriminative capability will bebe and a calibration graph will be plotted to show agreement between observed outcomes and the developed stratification model [59].

**Aim 4.** *Describe the impact of concussion on self and schooling over 1 year*. Descriptive statistics will be calculated for all variables that measure impact on self and schooling (Table 3). Bivariate analysis will be used to test whether PCS trajectory typology is associated with impact-on-self and -schooling outcomes using ANOVA for continuous variables and chi-square tests for categorical variables. Regression models will be built to assess whether associations hold after adjusting for other important covariates (e.g., age, sex, race, and education plan). Appropriate link functions for different types of outcomes will be used.

**Table 3:** Data-collection constructs, measures, and administration.

Construct (Aims)	Data or Measure	Variable Type	Time	Time Point (Source)
Demographics (Aims 1–4)	Age, sex, race, ethnicity, health insurance, zip code, area deprivation index	Categorical/Continuous	0	T1 (EHR)
Concussion characteristics (Aim 3)	Mechanism, Glasgow Coma Scale, loss of consciousness, amnesia, mental status changes, preexisting conditions, prior concussion(s), comprehensive health history	Categorical/Continuous	0	T1 (EHR)
Postconcussive symptoms (Aim 1)	Post-Concussion Symptom Scale (PCSS; 22 items)	Continuous	10 min	T1 (EHR-SR) T2–T6 (SR)
	PROMIS Pediatric v2.0–Fatigue (25 items)	Continuous	10 min	T1PI, T2 –T6 (SR)
Biomarkers (Aims 2, 3)	Salivary inflammatory cytokines and DHEA (puberty)	Continuous	2 min	T1–T6
	Salivary DNA	Continuous	3 min	T1
Impacts on self and schooling (Aim 4)	***Impact on self*:** Neurology Quality of Life (Neuro-QoL) v2.0–Pediatric Cognitive Function Short Form (8 items); Neuro-QoL v1.0–Pediatric Social Relations-Interaction with Peers Short Form (8 items)	Continuous	15 min	T1PI, T2–T6 (SR)
	***Impact on schooling***: Academic performance, no. missed school days, no. and types of missed classes, class grades mid/final-semester and end-of-grade report cards, days to return to learn/play, concussion and/or individualized education plan, CLASS-3 Adapted (45 items)	Continuous/Categorical	5 min	T1PI, T2–T6 (SR)

*Note*. T1 = at enrollment (within 7 days post-injury), no pre-injury data, T1PI = at enrollment, with pre-injury data; T2 = 1 mo, T3 = 3 mo, T4 = 6 mo, T5 = 9 mo, T6 = 12 mo; EHR = electronic health record; SR = self-report.

## Discussion

With the rate of concussions on the rise, our team of experts and this innovative project could help reduce the multifaceted burdens of concussion for well over a million children and their families each year. Early PCS diagnosis and access to evidence-based return-to-health and -school interventions are strongly linked to positive health and academic outcomes [7]. Yet clinical recognition tools to identify which children are at high risk for persistent PCS are lacking. The use of the NIH-SSM will provides the framework to develop a risk stratification model for PCS in children using novel PCS trajectory typologies, inflammatory biomarkers, demographics, and injury characteristics. Our model will enable timely identification of, and intervention for, children at highest risk for persistent PCS and poor health, academic, and social outcomes. Findings will pave the way for the development and testing of personalized symptom-management strategies to reduce disparities in outcomes and improve quality of life for children with concussion.

The strengths of this study include a large sample size of children between the ages of 11–17 years. The recruitment approach will include participants of both biological sexes and varying racial, ethnic, and socioeconomic backgrounds. This large and varied sample will allow for the inclusion of ample diversity to examine this complex and heterogeneous injury. Furthermore, our approach to participant retention will reduce barriers and burden for participants and ensure adequate sample size for our projected analyses.

Considering potential limitations to this study have allowed our team to put forth alternative strategies that would allow us to leverage all collected data. For example, the study of cytokine trajectories in relation to PCS trajectory typologies is a new area of research with many uncertainties. If the team is unable to detect distinct trajectory patterns in cytokines, piecewise GMM with three pieces will be used to capture the trajectory turning points. Instead of using distinct patterns in cytokine change, each child’s intercepts and slopes will be abstracted from GMM and compared among the PCS typologies. Additionally, while the models might not provide adequate sensitivity and specificity to distinguish associations between PCS trajectory typology and academic performance, the proposed study will yield valuable information about the impact of concussions on children’s everyday school and social lives.

## Conclusion

The novel use of trajectory analyses combined with NIH PROs and inflammatory biomarkers will advance symptom science in pediatric concussion. This research is a critical first step in developing personalized concussion symptom management in children. By enabling stratification of risk for PCS in children at the time of concussion diagnosis, our model will influence healthcare delivery, improve quality of life, and reduce the substantial health and socioeconomic burdens of concussions for children, families, schools, and healthcare systems.

## Supporting information

S1 Appendix Demographic, medical history, and psychosocial history instruments. (PDF)

S2 Appendix Concussion characteristics instrument. (PDF)

S3 Appendix Academic performance (CLASS-3 Adapted) instruments. (PDF)
