# Supplementary material for: A study protocol for risk stratification in children with concussion (RSiCC): Theoretical framework, design, and methods
Source: PLoS One. 2024 Jul 18;19(7):e0306399. doi: 10.1371/journal.pone.0306399 (PMC11257289; doi:10.1371/journal.pone.0306399)
Supplement: S1 Appendix — (PDF) [file pone.0306399.s001.pdf]

APPENDIX A

Demographics

**Age**	____________				
**Height in inches**	____________				
**Weight in pounds**	____________				
**BMI**	____________				
**Sex assigned at birth**	☐Female		☐Male		
**Ethnicity**	☐Non-Hispanic/Non-Latino		☐Hispanic/Latino		
**Race**	☐White	☐Black or African American	☐Asian		
	☐American Indian or Alaska Native	☐Native Hawaiian or Pacific Islander	☐Unknown or Not Reported		
**Health Insurance**	☐PPO	☐HMO	☐Self Pay	☐Military	☐Medicaid
**Area Deprivation Index***	☐1	☐2	☐3	☐4	☐5
	☐6	☐7	☐8	☐9	☐10

*Visit https://www.neighborhoodatlas.medicine.wisc.edu/mapping to determine the ADI based on the participant's home address

Past Medical History

1. I am healthy with no medical problems, past or present. ☐Yes ☐No

If "Yes", skip to question 3

2. I have had problems with (please select all that apply and list the problems):

☐Eye or eyesight ______

☐Ear, nose and throat ______

☐Thyroid or other endocrine ______

☐Heart or lung ______

☐Abdominal or urinary ______

☐Musculoskeletal ______

☐Nervous system ______

☐Other medical problems ______

3. In your medical history do you have the following conditions? For conditions that you do have, indicate whether you have had prior treatment for the condition.

Condition	*Prior Treatment?*	
☐Headache	☐Yes	☐No
☐Migraine	☐Yes	☐No
☐Family history of migraine		
☐Motion sickness	☐Yes	☐No
☐Learning disabilities and/or developmental disabilities	☐Yes	☐No
Please specify any learning and/or developmental disabilities: ______		
☐Chronic Fatigue	☐Yes	☐No
☐Sleep disorder	☐Yes	☐No
☐Attention Deficit Hyperactivity Disorder (ADHD/ADD)	☐Yes	☐No
☐Seizure disorder	☐Yes	☐No
☐Anxiety	☐Yes	☐No
☐Depression	☐Yes	☐No
☐Other psych-mental health disorder	☐Yes	☐No

Psycho-Social History

HOME		
Who lives at home with you? Please select all that apply.	☐Mom	☐Dad
	☐Parent's Partner	☐Sibling(s)
	☐Extended Family	☐Friends
	☐Other: ______________________	
Do you have your own room?	☐Yes	☐No

EDUCATION		
Please select your school type	☐Public School – Traditional	☐Public School – Charter
	☐Private School	☐Home school
	☐Virtual school	☐Other:
Please select your school schedule:	☐Traditional Calendar	☐Year-Round Calendar
Do you enjoy school?	☐Yes	☐No

EATING AND EXERCISE		
How many meals do you eat a day?	☐1	☐3
	☐2	☐4 or more
How many sugar sweetened beverages (SSB) do you drink a day? (e.g. non-diet soft drinks/sodas, flavored juice drinks, sports drinks, sweet tea, flavored coffee drinks, etc.)	☐0	☐3
	☐1	☐4 or more
	☐2	
How many caffeinated beverages do you drink in a day? (e.g.: coffee, espresso drinks, tea, energy drinks, some sodas, etc.)	☐0	☐3
	☐1	☐4 or more
	☐2	
Please select your general activity level:	☐Sedentary (little or no exercise)	
	☐Lightly Active (exercise/sports 1-3 days/week)	
	☐Moderately Active (exercise/sports 3-5 days/week)	
	☐Very Active (exercise/sports 6-7 days/week)	
	☐Extra Active (exercise/sports 2 or more times per day, or exercise/sports daily and a physical job)	

ACTIVITIES/SOCIAL		
On average do you feel like the activities you participate in are fun?	☐Yes	☐No
Do you participate in activities with friends?	☐In school<br>☐Outside of school<br>☐Both in school and outside of school<br>☐I do not participate in activities with friends	
What do you do ***most often*** with your free time?	☐Hang out with friends<br>☐Hang out with family<br>☐Hang out by myself – have “me” time	

SAFETY		
Do you always wear a seatbelt in the car?	☐Yes	☐No
Do you drive?	☐Yes	☐No
If you DO drive:		
What do you drive? Please select all that apply.	☐Car<br>☐Motorcycle<br>☐Motorized bike<br>☐Motorized scooter<br>☐Motorized wheeled toy<br>☐Golf cart	

Have you ever had a motor vehicle accident as the driver?	☐Yes	☐No
Have you sent a text message while driving?	☐Yes	☐No
Have you ever had more friends in the car than seat belts while driving?	☐Yes	☐No
Have you ever driven while tired?	☐Yes	☐No
Have you ever driving while stressed or upset?	☐Yes	☐No
Have you driven since you had a concussion?	☐Yes	☐No
