# Supplementary material for: A study protocol for risk stratification in children with concussion (RSiCC): Theoretical framework, design, and methods
Source: PLoS One. 2024 Jul 18;19(7):e0306399. doi: 10.1371/journal.pone.0306399 (PMC11257289; doi:10.1371/journal.pone.0306399)
Supplement: S2 Appendix — (PDF) [file pone.0306399.s002.pdf]

APPENDIX B

Concussion Characteristics

Date of injury:	MM/DD/YYYY	
Time of injury (if known) [24-hr format]:	HH:MM	
Cause of injury:	☐Assault<br>☐MVC (Motor vehicle collision)<br>☐Pedestrian vs. auto<br>☐Wheeled toy	☐Fall<br>☐Organized sport<br>☐Recreational sport<br>☐Other: ________
If cause is organized sport:		
What organized sport caused this injury? (if applicable)	☐Football<br>☐Soccer<br>☐Lacrosse<br>☐Basketball<br>☐Baseball/Softball<br>☐Cheer	☐Gymnastics<br>☐Track and Field<br>☐Wrestling<br>☐Ice Hockey<br>☐Volleyball<br>☐Other: ________
Did this injury occur during a game or during practice?	☐Game	☐Practice
If cause of injury is organized sport, recreational sport, or wheeled toy:		
Did the subject continue to play/practice in the same session of physical activity that caused the injury?	☐Yes	☐No
Patient wearing helmet at time of injury?	☐Yes ☐No	☐N/A
Location(s) of impact (check all that apply):	☐Frontal<br>☐Left Temporal<br>☐Right Temporal<br>☐Left Parietal<br>☐Right Parietal<br>☐Occipital	☐Neck<br>☐Face<br>☐Indirect Force<br>☐Multiple Impacts<br>☐Other: ________<br>☐Unsure
Retrograde amnesia? Are there any events just before the injury that the person has no memory of, even briefly?	☐Yes	☐No

Anterograde amnesia? Are there any events just after the injury that the person has no memory of, even briefly?	☐Yes	☐No
Loss of consciousness?	☐Yes	☐No

Early signs that appeared following the injury	☐Appears dazed or stunned	☐Is confused about events
	☐Answers questions slowly	☐Repeats questions
	☐Forgetful (recent info)	☐None of the above

Were seizures observed?	☐Yes	☐No

Seizure detail (***if applicable***):

Was the person dizzy immediately following injury?	☐Yes	☐No
Ate/Drank anything other than water within one hour following injury?	☐Yes	☐No
Emergency department referral at time of injury?	☐Yes	☐No

If ED referral:

Glasgow Coma Scale Score in ED:	☐13	☐14	☐15
Pupil Response:	☐Normal	☐Abnormal	
Imaging studies?	☐Yes	☐No	

If yes to imaging studies:

Imaging study type (check all that apply and provide notes):	☐CT:		☐EEG:	
	☐MRI:		☐Other:	

Is there evidence of an intercranial skull fracture?	☐Yes	☐No
Type of injury (from imaging):	☐SDH – subdural	☐FX – fracture
	☐EDH – epidural	☐NAICP – concussion
	☐SAH – subarachnoid	☐Other:
	☐DAI – diffuse axonal	
