# Supplementary material for: A study protocol for risk stratification in children with concussion (RSiCC): Theoretical framework, design, and methods
Source: PLoS One. 2024 Jul 18;19(7):e0306399. doi: 10.1371/journal.pone.0306399 (PMC11257289; doi:10.1371/journal.pone.0306399)
Supplement: S3 Appendix — (PDF) [file pone.0306399.s003.pdf]

APPENDIX C

NOTE: The items highlighted were incorporated as supplementary academic performance measures for this study and are not part of Gioia et al's CLASS-3.

Academic Performance – CLASS-3 Adapted – *Initial Study Visit*

General (pre-injury) school performance (Select ALL grades that apply):	☐A's	☐B's	☐C's	☐D's	☐E's/F's		
Since your concussion, how concerned are you about this injury affecting your school learning and performance? Please select ONE:	☐Not concerned<br>☐Mildly<br>☐Moderately<br>☐Very concerned						
Have you returned to learning since your concussion?	☐Yes			☐No			
If yes, how many days was it before you could return to learn?	☐0	☐1	☐2	☐3	☐4	☐5	☐6
How many missed class days have you had SINCE your injury?	☐0	☐1	☐2	☐3	☐4	☐5	☐6
Have you returned to play since your concussion?	☐Yes			☐No			
If yes, how many days was it before you could return to play?	☐0	☐1	☐2	☐3	☐4	☐5	☐6
What are your current grades (Select ALL grades that apply):	☐A's	☐B's	☐C's	☐D's	☐E's/F's		

Academic Performance – CLASS-3 Adapted – *Follow-up Study Visit*

Who at your school is planning or implementing your concussion-related supports? (Select ALL who apply)	☐School Nurse / Health Aide<br>☐Guidance Counselor<br>☐Principal / Asst. Principal<br>☐School Psychologist<br>☐Athletic Trainer<br>☐Homeroom Teacher<br>☐Special Education Teacher<br>☐Other Teacher<br>☐Coach<br>☐Other<br>☐No one (none of the above)
Since your last visit, how concerned are you about this injury affecting your school learning and performance? Please select ONE:	☐Not concerned<br>☐Mildly<br>☐Moderately<br>☐Very concerned
Have your grades been affected (worsened) since your last visit?	☐Yes ☐No

Think about the past few days and tell us whether the following school problems are worse because of your concussion.

	Not worse/Not a problem	A little worse	Somewhat worse	A lot worse
Difficulty taking notes	☐	☐	☐	☐
Difficulty understanding new material	☐	☐	☐	☐
In class, work taking longer	☐	☐	☐	☐
Homework taking longer	☐	☐	☐	☐
Difficulty studying for tests or quizzes	☐	☐	☐	☐
Trouble remembering what was studied	☐	☐	☐	☐
Trouble reading	☐	☐	☐	☐
Easily distracted during class work	☐	☐	☐	☐
Easily distracted during homework	☐	☐	☐	☐

	Not worse/Not a problem	A little worse	Somewhat worse	A lot worse
Headaches interfering with classwork	☐	☐	☐	☐
Headaches interfering with homework	☐	☐	☐	☐
Tiring easily during the school day	☐	☐	☐	☐
Tiring easily during homework	☐	☐	☐	☐
Easily bothered by lights/screens or noise	☐	☐	☐	☐

What is currently stressful or overwhelming for you because of your concussion? Indicate your level of stress.

	Not stressful	A little stressful	Moderately stressful	Very stressful
Missing time with friends and/or social activities	☐	☐	☐	☐
Not being allowed to play sports/recreation	☐	☐	☐	☐
Not having enough support from teachers	☐	☐	☐	☐
Not having enough support at home from parents/siblings	☐	☐	☐	☐
More stressed out/overwhelmed with the schoolwork piling up	☐	☐	☐	☐
Stressed out about your grades dropping	☐	☐	☐	☐

Because of your concussion, have you been having any NEW or WORSENING trouble in any of the following classes?

	No different	A little worse	Somewhat worse	A lot worse	Not taking
Math	☐	☐	☐	☐	☐
Reading/English/Language Arts	☐	☐	☐	☐	☐
Science	☐	☐	☐	☐	☐
History/Social Studies	☐	☐	☐	☐	☐
Foreign Language	☐	☐	☐	☐	☐

Please check one column for each of the following supports to indicate which supports you need and/or are receiving ***because of your concussion*.**

	Do you need it?			Do you have it?			
	No	Yes	I don't know	No	Yes, but not enough	Yes	I don't know
Shortened day	☐	☐	☐	☐	☐	☐	☐
Shorter classes	☐	☐	☐	☐	☐	☐	☐
Rest breaks	☐	☐	☐	☐	☐	☐	☐
Extra time to complete work	☐	☐	☐	☐	☐	☐	☐
No tests	☐	☐	☐	☐	☐	☐	☐
Modified tests (shorted length, more time, no screens, etc.)	☐	☐	☐	☐	☐	☐	☐
Current work reduced or waived	☐	☐	☐	☐	☐	☐	☐
Makeup work reduced or waived	☐	☐	☐	☐	☐	☐	☐
Coordinated plan for makeup work	☐	☐	☐	☐	☐	☐	☐
Other:	☐	☐	☐	☐	☐	☐	☐

Have you returned to learning since your concussion? ☐Yes ☐No

If yes, how many days was it before you could return to learn? ____________________

How many missed class days have you had SINCE your injury? ____________________

Have you returned to play since your concussion? ☐Yes ☐No

If yes, how many days was it before you could return to play? ____________________

What are your current grades (Select ALL grades that apply): ☐A's ☐B's ☐C's ☐D's ☐E's/F's
